# Editorial: Bridging knowledge to action in vitamin D supplementation

**DOI:** 10.3389/fnut.2026.1915845

**Published:** 2026-07-21

**Authors:** Manuela Chiavarini, Irene Giacchetta, Roberto Fabiani

**Affiliations:** 1 Department of Health Sciences (DSS), University of Florence, Florence, Italy; 2 Local Health Unit of Bologna, Department of Hospital Network, Hospital Management of Maggiore and Bellaria, Bologna, Italy; 3 Department of Chemistry, Biology and Biotechnology, University of Perugia, Perugia, Italy

**Keywords:** cardiometabolic health, implementation science, inflammation, mental health, precision nutrition, sleep, supplementation, vitamin D

Vitamin D supplementation stands at a translational crossroads. Its biological relevance is well-established, supplementation is accessible, and suboptimal vitamin D status remains common across clinical and population settings. From a public health perspective, addressing vitamin D deficiency requires integration of nutritional epidemiology, prevention strategies, and health promotion approaches, particularly in populations characterized by social, demographic, or clinical vulnerability. Yet the central challenge is no longer simply whether vitamin D matters, but how evidence can be converted into safe, targeted, and measurable action. The contributions to this Research Topic move the field beyond a generic “more vitamin D” message. They suggest that the future of supplementation lies in precision: identifying who is likely to benefit, which outcomes should be targeted, what dose and formulation are appropriate, and how efficacy and safety should be monitored.

A first theme emerging from the Research Topic is that vitamin D should be interpreted as a regulator of biological networks rather than as a single-purpose micronutrient. Tripathi and Carlberg provide mechanistic support for this view by identifying early vitamin D-responsive genes in human immune cells, including genes linked to antioxidant defense and redox homeostasis. This mechanistic layer is clinically meaningful because several contributions connect vitamin D status with inflammation-related phenotypes. Liu et al. reported that higher serum 25-hydroxyvitamin D concentrations were associated with lower prevalence of overactive bladder in females, with inflammatory biomarkers partly attenuating the association. Deng et al. extended this inflammatory perspective to intervention studies, showing that magnesium and vitamin D/E co-supplementation improved 25-hydroxyvitamin D status and reduced inflammatory markers in overweight or obese populations, while also emphasizing heterogeneity across trials. Together, these studies support a view of vitamin D action as context-dependent and biologically integrated with immune, inflammatory, and micronutrient networks.

A second theme is phenotypic heterogeneity. The articles do not support a one-size-fits-all model of supplementation; rather, they show that associations vary by sex, age, body composition, comorbidity, and vitamin D isoform. Sleep-related outcomes illustrate this point. Cai et al. summarized mechanistic and clinical evidence linking

vitamin D with sleep regulation through neuroendocrine, circadian, neurotransmitter, and neuroimmune pathways. Hung et al. then provided large-scale retrospective evidence that sustained vitamin D deficiency was associated with subsequent risk of obstructive sleep apnea, with stronger associations in women, younger adults, and overweight or obese individuals. These findings suggest that vitamin D assessment may be most informative when embedded in broader risk profiles rather than applied uniformly.

The same lesson emerges from mental health and cardiometabolic studies. Huang et al. found that vitamin D isoforms were differentially associated with depressive symptoms in U.S. adults, with vitamin D3 showing an inverse association and vitamin D2 showing an opposite pattern. Although cross-sectional, this observation challenges the tendency to treat “vitamin D” as a biologically homogeneous exposure. Cao, Xu et al.. reported sex- and age-specific relationships between vitamin D and serum uric acid in a large Chinese cohort, reinforcing the need for stratified interpretation. Zhang et al. examined hypertensive patients and linked vitamin D deficiency with adrenal, renal, and cardiovascular function-related indicators, while appropriately recognizing the limits of causal inference in this clinical context. Across these articles, vitamin D status functions less as an isolated laboratory value and more as a marker whose meaning depends on physiological and clinical background.

A third theme concerns intervention design. Translating knowledge into action requires more than identifying associations; it requires decisions about dose, duration, co-nutrients, delivery, and monitoring. Cao, Zhou et al. addressed this issue directly in a dose-response systematic review and meta-analysis in patients with diabetes mellitus and vitamin D deficiency. Their findings suggested that higher-dose supplementation, above 4,000 IU/day, may improve vitamin D status and glycemic control, while also underscoring the need to clarify long-term safety and risk-benefit balance (Cao, Zhou et al.) This is a crucial point for implementation: intensification should not be confused with personalization. Higher doses may be appropriate in selected settings, but they require a clear indication, defined goals, and biochemical safety surveillance.

The contribution by Sringari et al. adds another dimension to implementation by showing how *in-silico* modeling can be used to predict the effects of nutritional interventions on biochemical, growth, and body composition outcomes in Indian children. Although such models cannot replace clinical trials and require validation across populations, they can refine hypotheses, optimize intervention design, and prioritize resource-intensive studies. In this sense, computational modeling, co-supplementation strategies (Deng et al.), and dose-response evidence (Cao, Zhou et al.) all point toward a more deliberate implementation pipeline: from biological plausibility, to risk stratification, to intervention modeling, to pragmatic clinical evaluation.

At the same time, this Research Topic highlights why caution is essential. Several contributions are observational, cross-sectional, retrospective, *in-silico*, or proof-of-principle in design. These approaches are valuable for generating hypotheses, identifying subgroups, and clarifying mechanisms, but they cannot by themselves establish universal supplementation policies. The field therefore needs pragmatic randomized trials, longer follow-up, harmonized definitions of deficiency and sufficiency, careful comparison of vitamin D2 and D3, and standardized reporting of safety outcomes. Future studies should also integrate co-nutrient status, adiposity, sex, age, ethnicity, renal function, inflammatory profiles, and medication use into both trial design and clinical decision-making. Moreover, public health research should explore how socioeconomic conditions, health literacy, dietary patterns, and access to preventive services influence both vitamin D status and adherence to supplementation programs.

Bridging knowledge to action in vitamin D supplementation therefore requires a shift in emphasis. The goal is not indiscriminate supplementation or uniform high-dose treatment, but monitored personalization. In clinical practice, this means identifying groups at higher risk of deficiency or poorer outcomes, defining the intended endpoint of supplementation, selecting dose and formulation accordingly, and monitoring both response and safety. In public health, it means preserving simple and equitable strategies while recognizing that population-level messages must coexist with subgroup-specific recommendations.

The challenge ahead is not only biological but also organizational. Implementation science can support the translation of vitamin D evidence into feasible and scalable clinical and public health strategies. While personalized supplementation may optimize benefit-risk ratios, population-based approaches remain essential for groups at increased risk of deficiency and its consequences. Collectively, the articles in this Research Topic support a shift from universal approaches toward more targeted and evidence-informed vitamin D strategies. The priority is not broader supplementation *per se*, but the development of measurable, context-sensitive interventions that optimize clinical benefit, safety, and public health impact through risk stratification, monitoring, and effective implementation.

